# Controllable synthesis of fluorescent silver nanoparticles with different length oligonucleotides

**DOI:** 10.1049/nbt2.12049

**Published:** 2021-03-22

**Authors:** Wenhui Bao, Jun Ai, Lu Ga

**Affiliations:** ^1^ College of Chemistry and Environmental Science Inner Mongolia Key Laboratory of Environmental Chemistry Inner Mongolia Normal University Hohhot China; ^2^ College of Pharmacy Inner Mongolia Medical University Hohhot China

## Abstract

Silver nanomaterials have become important research topics in recent years. As a new type of fluorescent material, silver nanomaterials have been applied to fluorescent sensors, bioimaging and materials targeting cancer cells. Here, an approach to the oligonucleotide‐templated controllable formation of fluorescent Ag nanomaterials is reported. In this experiment, silver nanoparticles (NPs) were synthesised from oligonucleotides chains, sodium borohydride (NaBH_4_) and silver nitrate (AgNO_3_) by changing the molar ratio of DNA to sodium borohydride (NaBH_4_) and silver nitrate (AgNO_3_). Fluorescent assay and transmission electron microscopy were used to characterise the silver NPs. The optimal selection of DNA chains with different lengths as templates for the synthesis of silver NPs was found. This work successfully develops the capping oligonucleotides scaffolds of silver nanoclusters.

## 1 INTRODUCTION

The structure of DNA consists of a pair of polynucleotide chains coiled around the central axis. Sugar‐phosphoric acid chains are outside the helical structure and base groups are inward, forming a fairly stable structure [1]. Based on the classical Watson–Crick pair interaction, that is, A pair and T, G pair and C, single‐stranded DNA molecules can be hybridised to form double‐stranded DNA [2]. Usually, the resulting helix is 0.34 nm long and 2 nm wide. Therefore, both DNA structure and nanomaterials are of the same size level.

Natural DNA can easily be extracted from bacteria, animals and viruses as genetic information for storage, transmission and translation. On the other hand, so far, based on a simple four‐letter alphabet, DNA can easily be synthesised by automated methods and polymerase chain reaction with arbitrary sequences or lengths, and the cost is relatively low. Compared with other common biological molecules such as proteins or peptides, DNA is an easily obtained biopolymer suitable for nano‐template manufacturing because of its relative stability, programmable size and predesigned properties. More important, DNA has abundant chemical functions, and the state allows it to combine various metal ions to produce various nanomaterials.

Recent advances have demonstrated that DNA metallisation can produce nano‐metals with unique conductivity, which are widely used in catalysis, sensing, electronic energy conversion, storage and biotechnology.

Chemical reduction of metal cations is the most widely used method for DNA metallisation, which was first proposed by Braun's team. For this, ions are then selectively located on the DNA profile. In subsequent reductions, 12‐mm‐long conductive silver nanowires were used to produce electrodes 100 nm wide on water surfaces. Measurement of this metal ion by the two‐terminal electrical method fully shows the electric transport ability of silver [3]. Although there are more or less shortcomings on DNA bridges, this pioneering work has made great progress in fabricating nanowires metallised by DNA molecules.

Silver nanomaterials refer to the use of state‐of‐the‐art nanotechnology to produce silver products in the range of 1–100 nm, which is the size of a single atom up to dozens of atoms [4]; this means that its surface area is large, its atoms participate in a large number of chemical reactions, and the atoms will show better reactivity when they are not bonded. In addition, nano‐size silver materials can exhibit some properties of both metal nanomaterials and bulk metals [5, 6, 7, 8, 9].

Because of the small size of silver nanoparticles (NPs), their area will gradually increase in the chemical reaction process.

There are obvious differences in the electronic states, bonding states and internal states of silver NPs [10, 11, 12]. The incomplete coordination of atoms on the surface leads to an increase in their active sites, which greatly improves their catalytic performance.

Because of the special properties of silver nanomaterials, they have broad application prospects in catalysis, sensing, optics, electricity and medicine.

Moreover, silver nanomaterials have excellent antioxidant properties, plasticity, stability and conductivity while retaining the traditional properties of nanomaterials, and they have a wide range of applications in chemical catalysis and detection [13].

Silver metal has excellent photoelectric properties [14], so the preparation of its nano‐materials has been a hot research topic in the field of new materials for many years. The application range of silver nano‐materials is becoming increasingly wider.

The combination of fluorescent NPs and molecular species can enable us to attempt to use a variety of technologies to understand medical biological processes more comprehensively. It has potential and promising applications in related biological analysis and biomedical diagnosis. NPs will form various morphologies on a DNA template; whether DNA will act as a template during the growth of NPs is the main basis for research.

## 2 EXPERIMENTAL SECTION

### 2.1 Reagents and chemicals

The oligonucleotides (Table S1) were synthesised by Shanghai Sangon Biotechnology Co., Ltd. Phosphate‐buffered saline (PBS) and sodium borohydride (NaBH_4_) were bought from Sheng'ao Chemical Reagent Co., Ltd. Silver nitrate (AgNO_3_) was obtained from Kaima Reagent Co., Ltd. All chemicals were used as received without further purification. Milli‐Q water was used throughout.

### 2.2 Synthesis of oligonucleotides‐templated fluorescent Ag NPs

After centrifuging the oligonucleotide primer at 5000 rpm for 15 min, a PBS buffer solution (pH 7.4) was added to it to prepare a 100‐μM solution, which was subjected to denaturation treatment in a water bath at 95°C for 30 min, followed by annealing treatment for 1 h. PBS buffer solution (10 mM, 300 μL) was added to the oligonucleotide solution (25 mM, 10 μL), shaken to make it react fully and mixed well. Then AgNO3 solution (250 μM, 100 μL) was added to the mixed solution and put in an ice bath to react for 30 min. An NaBH4 solution (50 μM, 100 μL) was added to the mixed solution and vigorously oscillated for 1 min, and the mixed solution was finally sonicated for 10 min to obtain DNA‐Ag NPs.

### 2.3 Apparatus

All fluorescence spectra were recorded on a Hitachi F‐4600 fluorescence spectrophotometer. Transmission electron microscopy (TEM) images were obtained on a JEOL‐2100F high‐resolution transmission electron microscope operated at an accelerating voltage of 200 kV. Fourier transform infrared spectroscopy (FT‐IR) spectra were recorded in the wavelength range of 4000–500 cm^−1^ with a Nicolet Avatar 360 FT‐IR spectrophotometer.

## 3 RESULTS AND DISCUSSION

### 3.1 Characterisation of oligonucleotides‐Ag NPs synthesised under optimal conditions

The fluorescence excitation/emission wavelength of silver NPs is shown in Figure S1, which shows that the excitation peak of A‐Ag NPs is 286 nm and the emission peak is 390 nm; C‐Ag NPs is excited at 285 nm and emitted at 390 nm; G‐Ag NPs is excited at 281 nm and emitted at 388 nm; and the T‐Ag NPs is excited at 291 nm and emitted at 393 nm.

According to Figure S2, the excitation peak of M‐Ag NPs is 283 nm and the emission peak is 383 nm. N‐Ag NPs is excited at 284 nm and emitted at 390 nm. The excitation peak of O‐Ag NPs is 290 nm and the emission peak is 390 nm.

**FIGURE 1:**
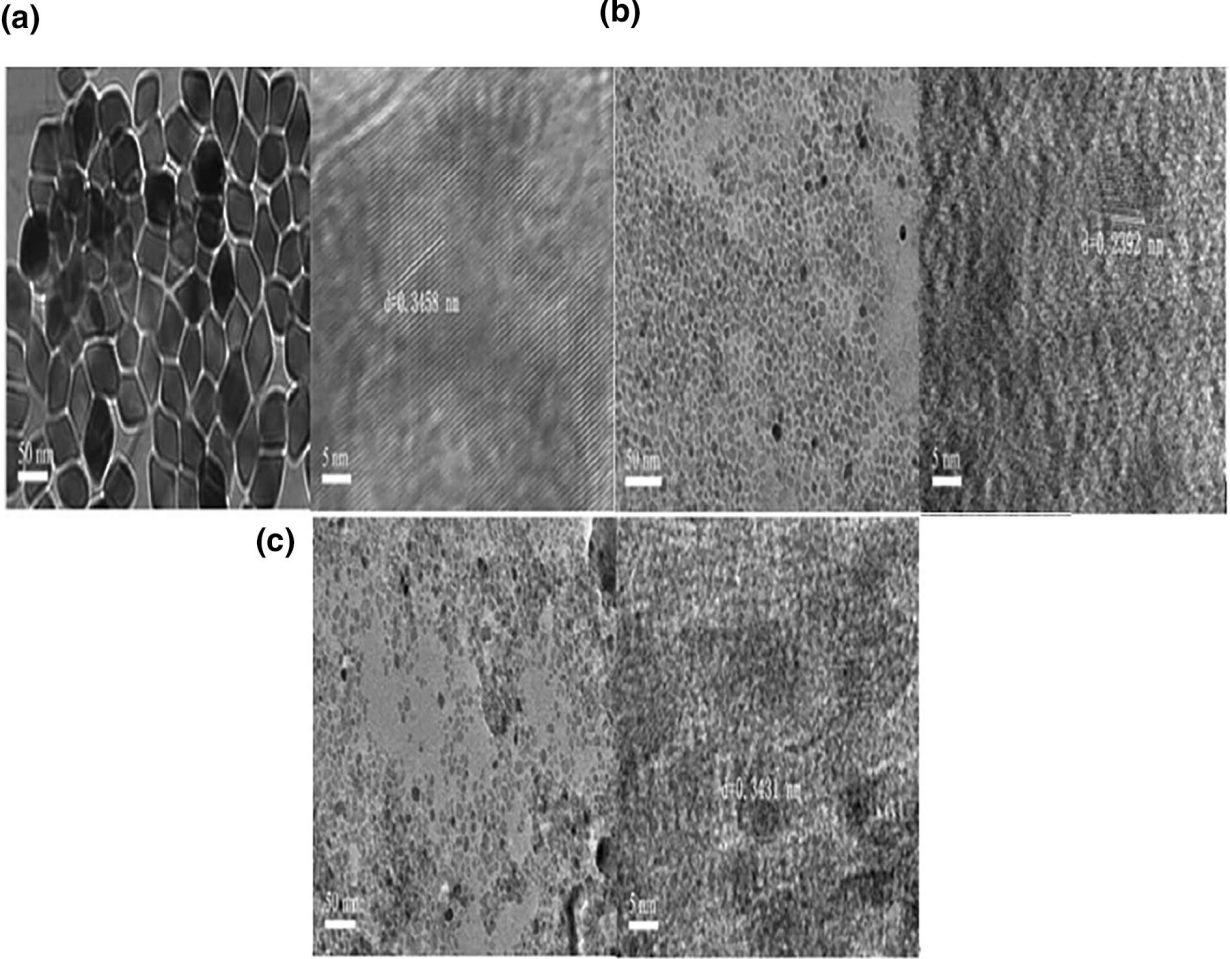
Transmission electron microscopy image of nanoparticles: M‐Ag (a),N‐Ag (b), and O‐Ag (c) and high‐resolution transmission electron microscopy image of M‐Ag (a), N‐Ag (b), O‐Ag (c)

Figure 1 shows that the particle size of M‐Ag NPs is about 43.5 nm, its morphology is rhombic, the particle size is uniform, and its lattice spacing *d* = 0.34 nm. The particle size of N‐Ag NPs is about 10.9 nm and its morphology is spherical with a uniform particle size. Its lattice spacing *d* = 0.23 nm. The particle size of O‐Ag NPs is about 8.7 nm and its morphology is spherical with a uniform particle size. Its lattice spacing *d* = 0.34 nm.

**FIGURE 2:**
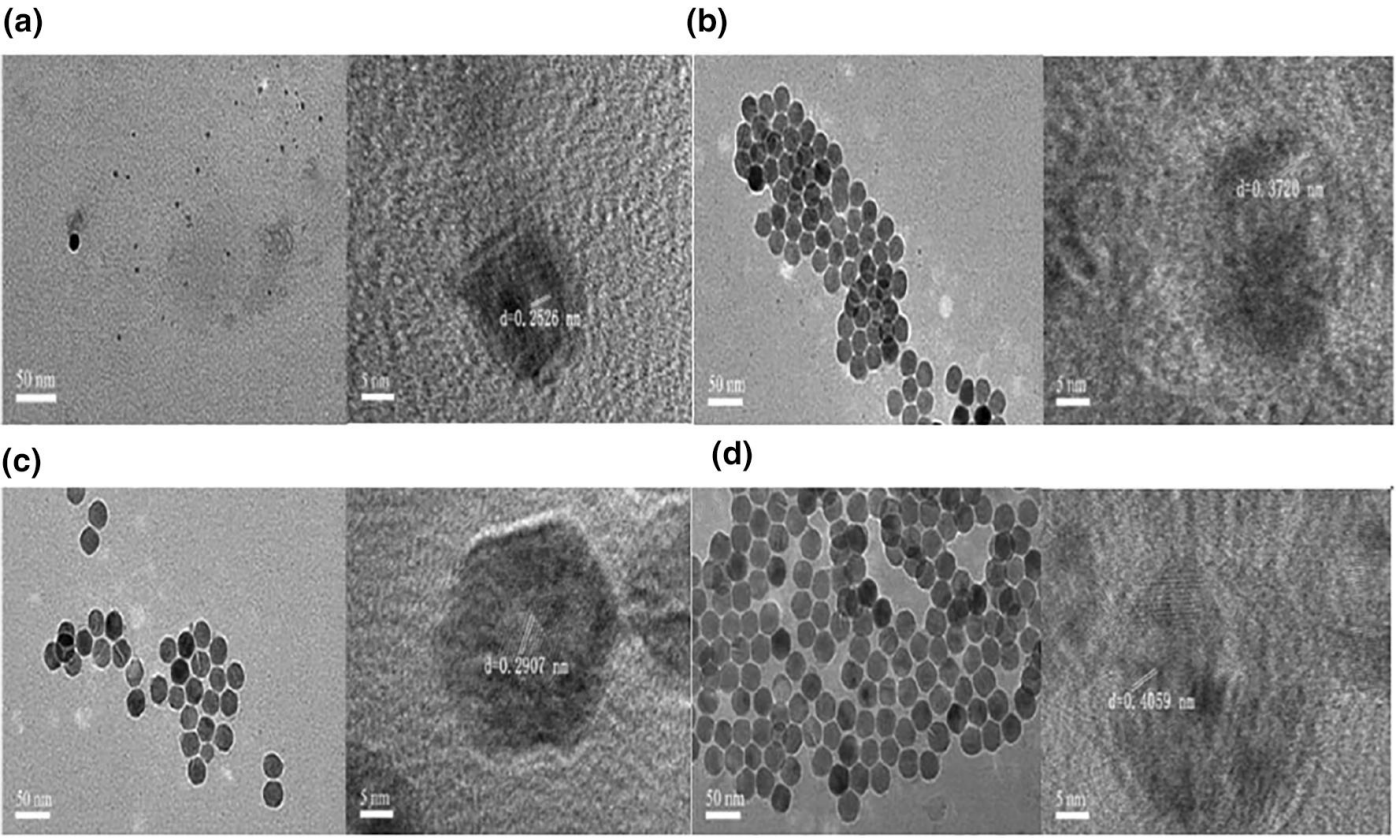
Transmission electron microscopy image of nanoparticles: A‐Ag (a), C‐Ag (b), G‐Ag (c) and T‐Ag (d)

Figure 2 shows that the particle size of A‐Ag NPs is about 4.4 nm, its morphology is spherical, the particle size is uniform, and its lattice spacing *d* = 0.25 nm. The particle size of C‐Ag NPs is about 21.7 nm. Its morphology is spherical with a uniform particle size. Its lattice spacing *d* = 0.3720 nm. The particle size of G‐Ag NPs is about 23.9 nm. Its morphology is spherical. It has a uniform particle size. Its lattice spacing *d* = 0.29 nm. The particle size of T‐Ag NPs is about 23.9 nm and its morphology is spherical with a uniform particle size. Its lattice spacing *d* = 0.4059 nm.

### 3.2 Optimising reaction conditions for synthesis of DNA‐Ag NPs

**FIGURE 3:**
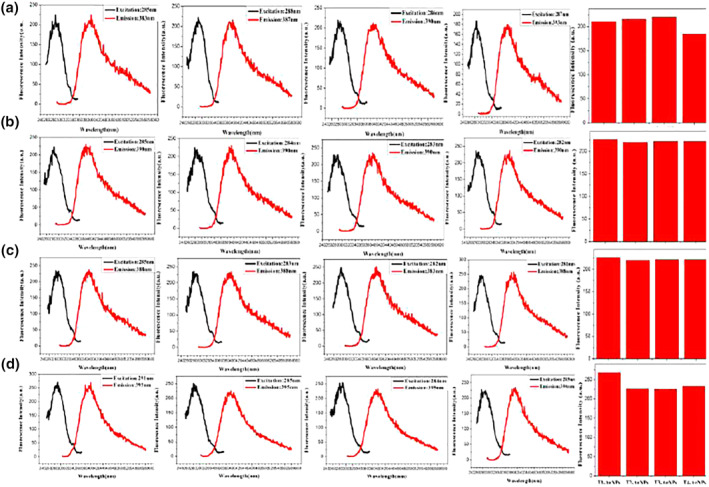
Fluorescence spectra of nanoparticles: A‐Ag (a), C‐Ag (b), G‐Ag (c) and T‐Ag (d)

When the reaction environment of synthesising oligonucleotides‐Ag NPs remains unchanged, different lengths of oligonucleotide sequences are used as templates for synthesising Ag NPs. Figure 3 shows that the emission peaks of Ag NPs synthesised with oligonucleotides as template are redshifted. By comparing the fluorescence intensity of Ag NPs protected by oligonucleotides strands of different lengths, A20, C12, G24 and T12 are selected as the optimal template for Ag NP synthesis.

**FIGURE 4:**
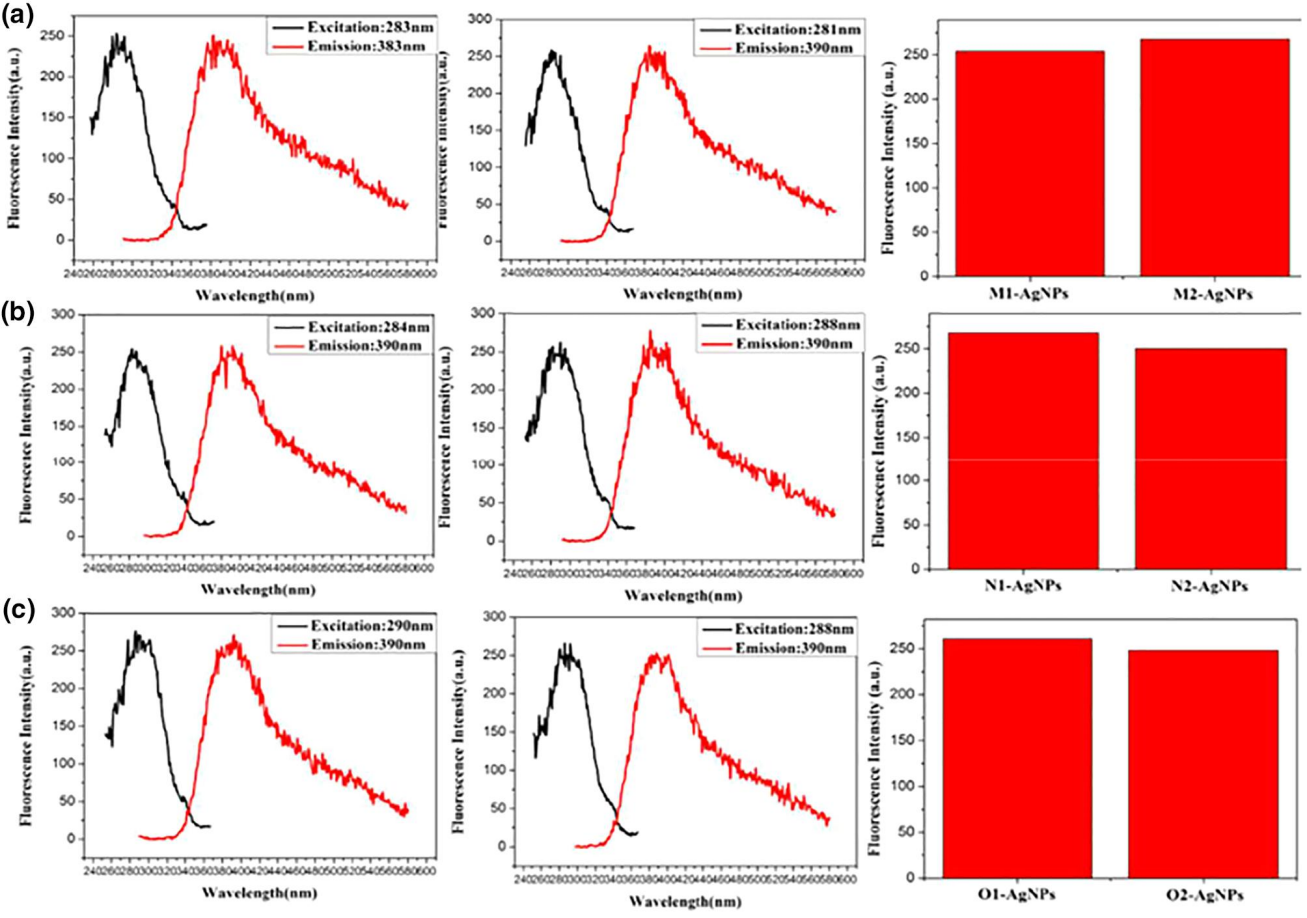
Fluorescence spectra of nanoparticles: M‐Ag (a), N‐Ag (b), and O‐Ag (c)

With the condition that the reaction environment for the synthesis of oligonucleotide‐Ag NPs does not change, the complementary oligonucleotide sequence is used as a template to synthesise Ag NPs, and its influence on the fluorescence performance of the synthesised Ag NPs is explored. Figure 4 shows that the emission peaks of Ag NPs synthesised using complementary oligonucleotides sequences as templates are redshifted. By comparing the fluorescence intensity of Ag NPs protected by complementary oligonucleotides sequences, M1, N1 and O1 are selected as the optimal templates for the synthesis of Ag NPs.

**FIGURE 5:**
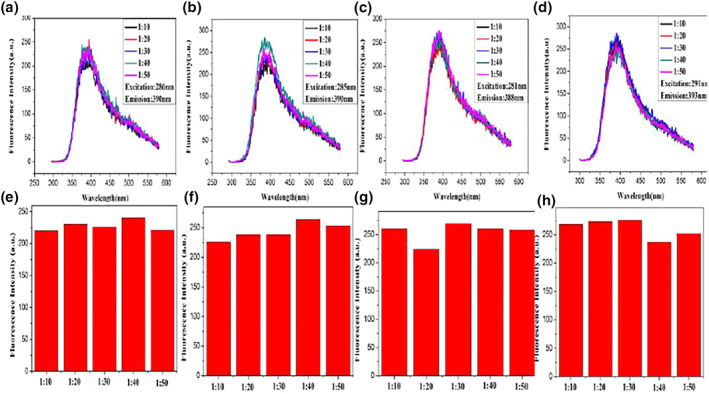
Fluorescence spectra of the Ag nanoparticles with different. molar ratios of AgNO3/oligonucleotides

With no change in other reaction conditions, only the molar ratios of oligonucleotides and AgNO3 were changed to 1:20, 1:30, 1:40 and 1:50, respectively. A series of samples were prepared and their fluorescence was measured to explore the effect of the molar ratios of oligonucleotides and AgNO3 on the fluorescence properties of Ag NPs. Figure 5 compares the fluorescence intensity of Ag NPs protected by oligonucleotide (A20) chains with different molar ratios; the optimum molar ratio is 1:40 (Figure S2, A, E). The fluorescence intensity of Ag NPs protected by oligonucleotide (C12) chains with different molar ratios was determined to be the optimum molar ratio of 1:40 (Figure S2B, F).

The fluorescence intensity of Ag NPs protected by different molar ratios of oligonucleotide (G24) chains was compared, and the optimum molar ratio was 1:30 (Figure S2C, G). The fluorescence intensity of Ag NPs protected by different molar ratios of oligonucleotides (T12) chains was determined, and the optimum molar ratio was 1:30 (Figure S2D, H).

**FIGURE 6:**
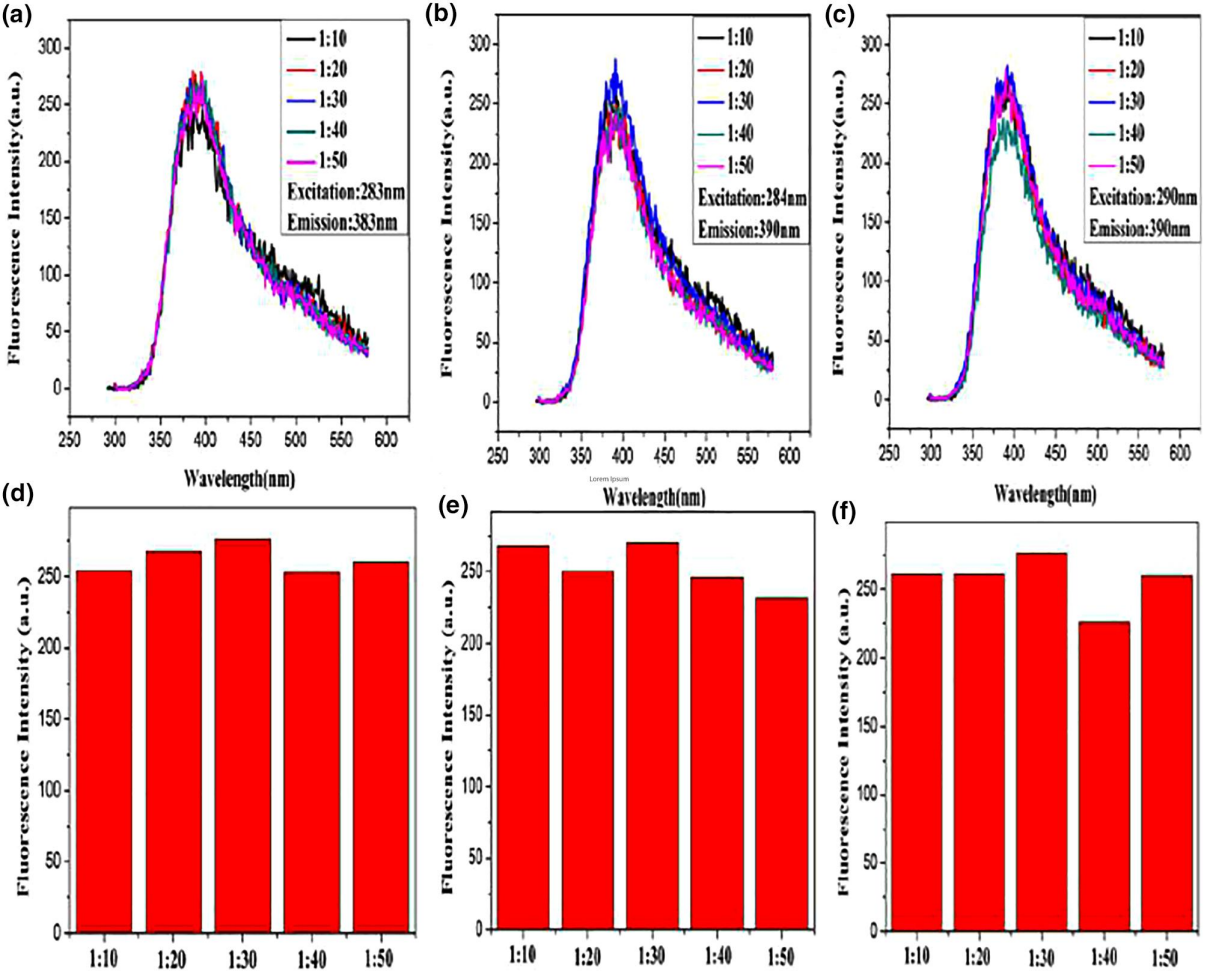
Fluorescence spectra of Ag nanoparticles with different molar ratios of AgNO3/oligonucleotides

Change only the molar ratios of concentration oligonucleotides and AgNO_3_ were changed to 1:20, 1:30, 1:40 and 1:50, respectively. A series of samples were prepared and their fluorescence was measured to explore the effect of the molar ratios of concentration oligonucleotides and AgNO_3_ on the fluorescence properties of Ag NPs. Figure 6 shows that the fluorescence intensity of Ag NPs protected by different molar ratios of oligonucleotides (M1) chains was compared, and the optimum molar ratio was 1:30 (Figure 6A, D). The fluorescence intensity of Ag NPs protected by oligonucleotide (N1) chains with different molar ratios was used to select the optimum molar ratio of 1:30 (Figure 6B, E). The fluorescence intensity of Ag NPs protected by oligonucleotide (O1) chains with different molar ratios was compared, and the optimum molar ratio was 1:30 (Figure 6C, F).

When the other reaction conditions were unchanged and the optimum molar ratio of C oligonucleotides to CAgNO3 was reached, only the molar ratio of C oligonucleotides to C(NaBH4) was changed to 1:1, 1:2, 1:3 and 1:5, respectively. A series of samples were prepared and characterised by fluorescence, and their effects on the fluorescence properties of Ag NPs were explored. In Figure S3, the optimum molar ratio of C oligonucleotides to C(NaBH4) is 1:1 in a comparison of the fluorescence intensity of Ag NPs protected by oligonucleotide (A20) chains (Figure S3A, E). Comparing the fluorescence intensity of oligonucleotide (C12) chain‐protected Ag NPs, the optimum molar ratio of C oligonucleotides to C(NaBH4) was 1:1 (Figure S3 B, F). The fluorescence spectra of oligonucleotide (G24) chain‐protected Ag NPs were compared, and the optimum molar ratio of C oligonucleotides to C(NaBH4) was 1:3 (Figure S3 C, G). The optimum molar ratio of cDNA to C(NaBH4) was 1:1 (Figure S3D, (h) in a comparison of the fluorescence intensity of Ag NPs protected by DNA (T12) chains.

When the other reaction conditions were unchanged and the optimum molar ratio of C oligonucleotides to CAgNO3 was reached, only the molar ratio of C oligonucleotides to C(NaBH4) was changed to 1:1, 1:2, 1:3 and 1:5, respectively. A series of samples were prepared and characterised by fluorescence, and their effects on the fluorescence properties of Ag NPs were explored. Figure S4 shows that the optimum molar ratio of C oligonucleotides to C(NaBH4) is 1:1 by comparing the fluorescence intensity of Ag NPs protected by oligonucleotides (MI) chains (Figure S4,D). In a comparison of the fluorescence intensity of oligonucleotides (N1) chain‐protected Ag NPs, the optimum molar ratio of C oligonucleotides to C(NaBH4) was 1:3. Figure S4B, E compares the fluorescence spectra of oligonucleotides (O1) chain‐protected Ag NPs, and the optimum molar ratio of C oligonucleotides to C(NaBH4) was 1:3 (Figure S4C, F).

In the absence of any change in other reaction conditions, oligonucleotides was not added as a template in the synthesis of Ag NPs, and the effect of oligonucleotide as a template on the synthesis of Ag NPs was discussed. Figure S5A shows that the fluorescence intensity of Ag NPs synthesised without a template oligonucleotide is significantly reduced. At the same time, under the same reaction conditions, NaBH4 was not added as a reducing agent during the synthesis. The effect of NaBH4 as a reducing agent on the synthesis of Ag NPs was discussed. Figure S5B shows that the fluorescence intensity of the synthesised sample is significantly reduced when NaBH4 is not added.

## 4 CONCLUSIONS

Oligonucleotide‐Ag NPs were successfully prepared in this study, and the optimum conditions for synthesis of oligonucleotide‐Ag NPs were obtained by adjusting the molar ratio of reactants. The roles of oligonucleotide and NaBH4 in the synthesis of oligonucleotide‐Ag NPs were discussed. The development of silver nanomaterials in various fields needs further research. Silver nanomaterials can be treated with other composite materials, and their applications in various fields can be increased over a wide range.

## Supporting information

Supplementary Material
