# Supplementary material for: Controllable synthesis of fluorescent silver nanoparticles with different length oligonucleotides
Source: IET Nanobiotechnol. 2021 Mar 22;15(5):512–8. doi: 10.1049/nbt2.12049 (PMC8675809; doi:10.1049/nbt2.12049)
Supplement: Supplementary file 1 — Supplementary Material [file NBT2-15-512-s001.docx]

Supporting Online Material for

**Controllable synthesis of fluorescent silver nanoparticles using oligonucleotides chains of different lengths as templates**

*Wenhui Bao^§^，Jun Ai^§，† *^, Lu Ga* *^‡*^*

*^§^College of Chemistry and Enviromental Science, Inner Mongolia Normal University, 81 Zhaowudalu, Hohhot 010022, China.*

*^‡^* *College of Pharmacy, Inner Mongolia Medical University, Jinchuankaifaqu, Hohhot 010110, People’s Republic of China.*

*^†^Inner Mongolia Key Laboratory of Environmental Chemistry, Inner Mongolia Normal University, 81 Zhaowudalu, Hohhot 010022, China.*

**Table S1** Oligonucleotides Primer Sequence List as Templat

**
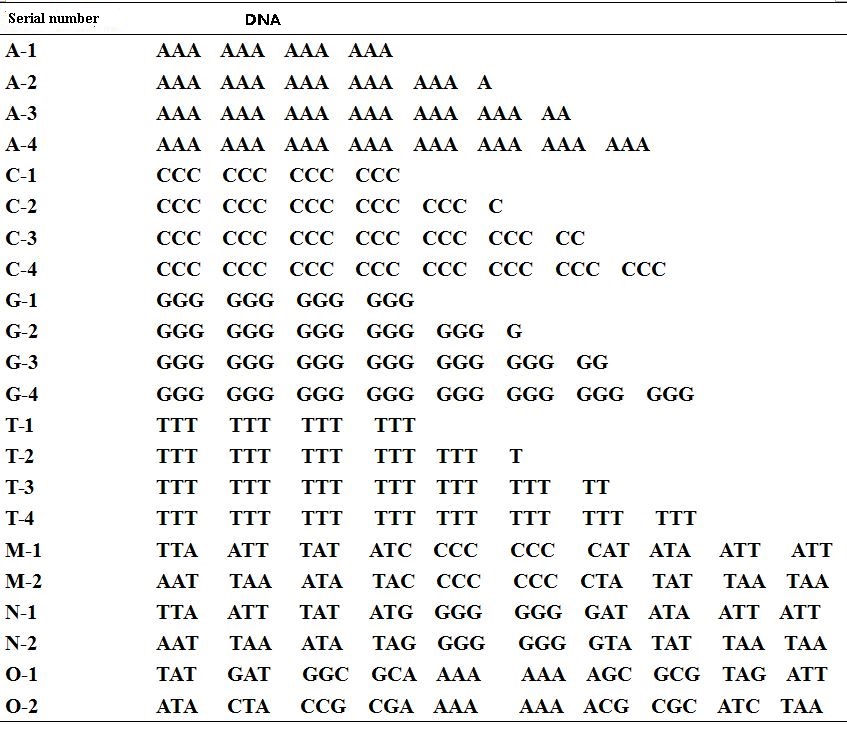
**


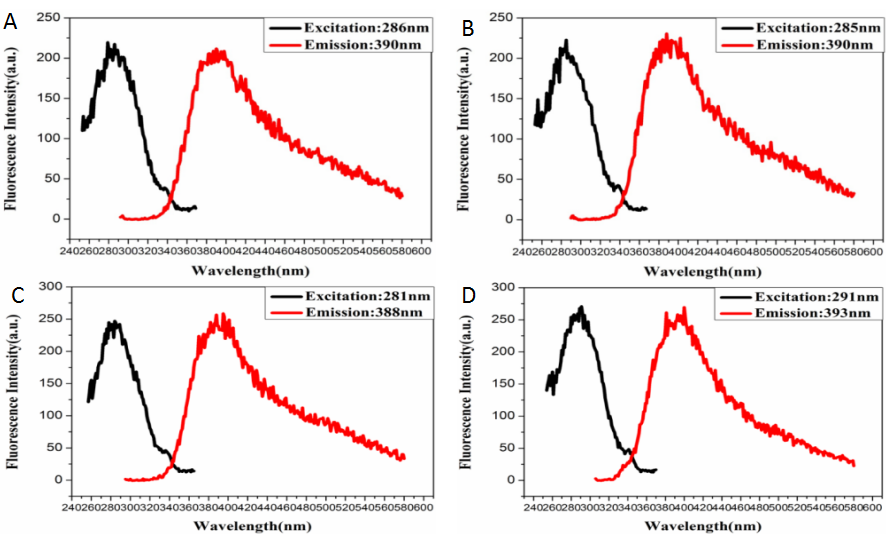


Fig.S1 Fluorescence spectra of A-Ag NPs(A),C-Ag NPs(B),G-Ag NPs(C) and T-Ag NPs(D).


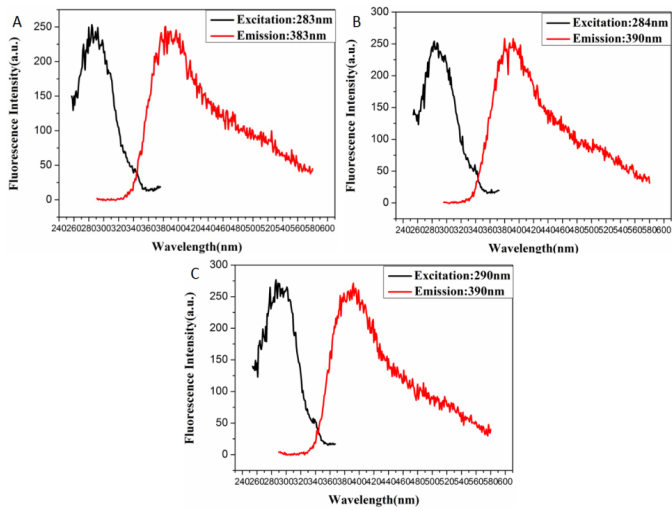


Fig.S2 Fluorescence spectra of M-Ag NPs(A),N-Ag NPs(B),and O-Ag NPs(C).


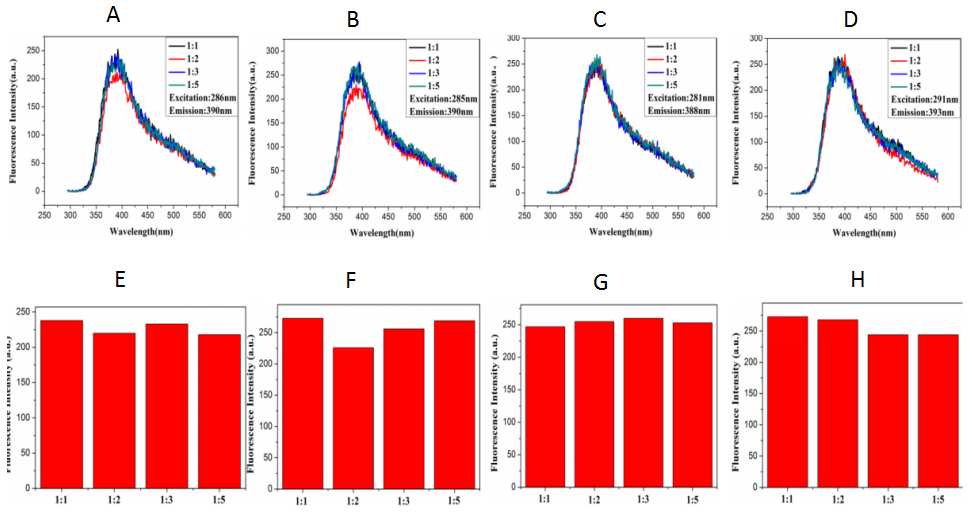
 Fig.S3 Fluorescence spectra of the different molar ratios between oligonucleotides and NaBH_4_.


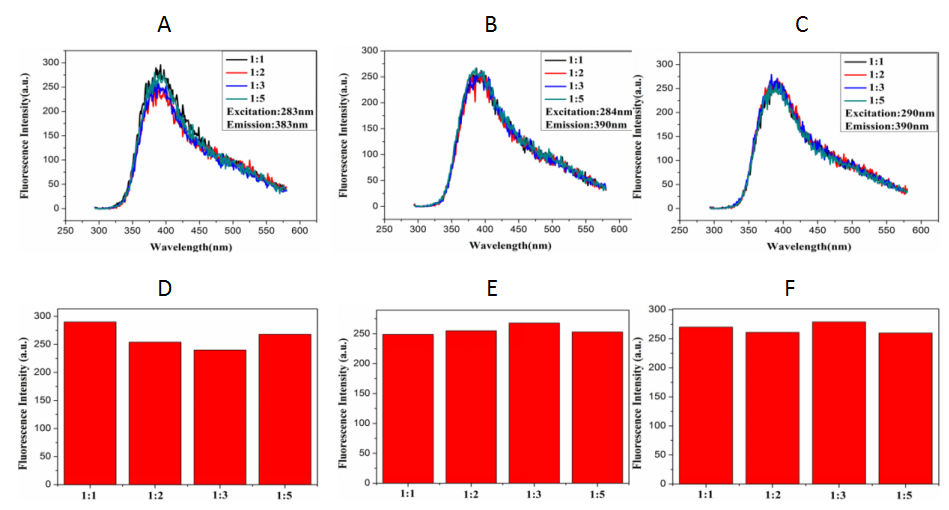
 Fig. S4 Fluorescence spectra of the different molar ratios between oligonucleotides and NaBH_4_.


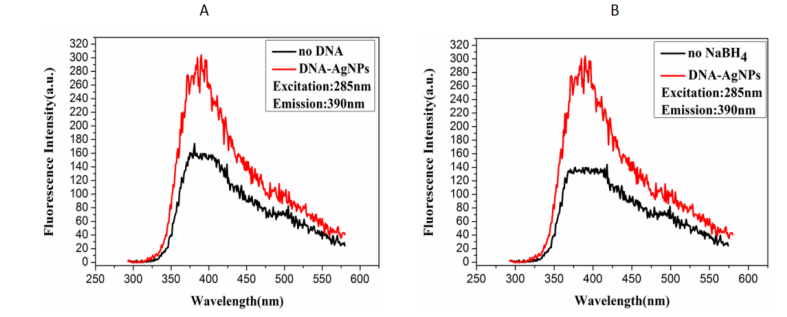
 Fig.S5 Fluorescence spectra of without oligonucleotide (A) and without NaBH_4_ (B).
